# Selection and connection: the impact of internet use on the depression of Chinese older adults

**DOI:** 10.3389/fpubh.2025.1524276

**Published:** 2025-03-19

**Authors:** Kaishan Jiao, Yue Sun

**Affiliations:** School of Ethnology and Sociology, Minzu University of China, Beijing, China

**Keywords:** internet, social networks, depression, mental health, older adults, China

## Abstract

**Introduction:**

Internet usage has significant effects on the mental health of older adults, particularly in reducing depression levels. However, its impact may differ based on factors such as internet usage frequency, purposes, gender, and urban-rural residence. This study aims to examine the differential effects of internet use on depression among older adults, exploring the mediating role of social networks in these relationships.

**Methods:**

Based on panel data from the 2018 and 2020 China Longitudinal Aging Social Survey, a fixed-effects model was employed to systematically analyze the impact of internet use (including whether they use the internet, frequency of use, and purposes of use) on the mental health of older adults. The mediating role of social networks (family networks and friend networks) was tested using the Bootstrap method.

**Results:**

The study found that internet use significantly reduces depression levels among older adults (coefficient = −0.440, *p* < 0.001), with notable differences in mental health benefits based on gender and urban-rural residence. Both family networks and friend networks mediate the relationship between internet use and depression. Specifically, older adults who use the internet frequently or for interpersonal communication tend to expand their social networks, which in turn improves their mental health. Family networks exhibit a stronger mediating effect compared to friend networks. However, the positive effects of leisure and information-seeking internet activities are partially offset by the reduction in social network size.

**Discussion:**

This study highlights the complex relationship between internet use, social networks, and mental health in older adults. Significant gender and urban-rural differences exist in the impact of internet use on depression among older adults, indicating the need for tailored interventions. The findings also emphasize the importance of promoting internet use for social purposes while being mindful of the potential negative impacts of excessive digital engagement among older adults, particularly in rural areas. Additionally, social networks, especially friend networks, play an important role in enhancing the mental health benefits of internet use for older adults.

## 1 Introduction

In the 21st century, we face dual challenges: aging and informatization, both of which are shaping our social reality. According to the 54th Statistical Report on Internet Development in China released by the China Internet Network Information Center (CNNIC), as of June 2024, the number of internet users in China has reached nearly 1.1 billion (1.09967 billion), with an internet penetration rate of 78%. The report also indicates that the “silver-haired” demographic is a significant source of new internet users, with individuals aged 60 and above accounting for 20.8% of new internet users, highlighting the further penetration of the internet into the older adult population. Meanwhile, the Blue Book of the “Report on the Development of National Mental Health in China (2019–2020)” reveals that nearly one-third of older adults in China experience depression, making it one of the most common psychological symptoms among the older adult population. The report of the 20th National Congress of the Communist Party of China emphasizes the need to advance the Healthy China initiative, deepen the Healthy China Action, and implement a national strategy to actively respond to population aging, with promoting the mental health of the older adults being a core component of the Healthy China strategy (1). Against this backdrop, in an era where the “silver wave” and internet popularization are concurrently advancing, the relationship between internet use and the health of older adults, particularly their mental health, has garnered widespread attention and intense discussion among scholars both domestically and internationally.

The rapid development of information technology has enabled an increasing number of older adults to enjoy more convenient information transmission and a diverse range of life services through the internet, greatly enriching their spiritual and cultural lives (2). This phenomenon has led to a growing body of research exploring the impact of internet use on the mental health of older adults. Two predominant theoretical perspectives have emerged in this area: the Network Enhancement Benefit Theory and the Presence Substitution Benefit Theory. According to the “Network Enhancement Benefit Theory,” internet use helps older adults expand their social networks, strengthen social connections, and alleviate feelings of loneliness and depression, thereby improving their mental health. Consequently, most studies, both domestically and internationally, exploring the relationship between internet use and the mental health of older adults suggest that internet use can further expand the social networks of older adults, enhance their social connections, and thus alleviate feelings of loneliness and depression, having a positive impact on their mental health (3–8). However, at the same time, excessive internet use can lead to “digital addiction,” causing older adults to isolate themselves from the real world, resulting in a decline in social skills and potentially more severe mental health issues. This aligns with the “Presence Substitution Benefit Theory,” which posits that excessive internet use encroaches upon time for offline activities, reducing social participation and interpersonal interactions, thereby exerting negative effects on mental health (9, 10). Furthermore, some scholars have pointed out that the relationship between internet use and the health status of older adults may be influenced by the specific content of their usage (11, 12).

Given that the internet is a mixed blessing, with both positive and some negative effects, understanding in more detail for whom it is beneficial and how it can be beneficially used would support evidence-based guidelines for using modern information technology. This is particularly important for older adults, as the internet holds the potential to enhance their social participation and mental wellbeing. However, there are still many unanswered questions. What effect does the internet actually have on the mental health of older adults? How does it exert this effect? And what does it mean for older adults in urban vs. rural areas and for different genders? Clarifying these issues can help us better understand the impact of the internet on the mental health of older adults, with the aim of establishing a safe, positive, and supportive online community. This would enable older adults to better utilize modern information technology, better meet their mental health needs, enhance their ability to participate in the digital society, and improve their quality of life in their later years.

## 2 Literature review

### 2.1 Analysis of mental health and its influencing factors in older adults

Health is a multifaceted concept encompassing various dimensions such as physical, functional, cognitive, and psychological health (13). Among these, psychological health is a key factor influencing the wellbeing of older adults and is a focal point in China's strategic response to population aging (14). A review of previous studies on the factors affecting the psychological health of older adults reveals that it is influenced by individual, family, and social factors. Generally, among individual factors, older adults who are older, living in rural areas, female, living alone, without a spouse, with low educational attainment, or suffering from chronic diseases are more likely to have weaker psychological health compared to other older adult groups (15–19). As age increases, the decline in physical function, increased incidence of chronic diseases, and major life changes such as widowhood, retirement, and living alone make them more susceptible to depressive moods (20). In terms of family factors, intergenerational support from children significantly impacts the psychological health of older adults. Studies indicate that emotional and financial support from children can positively influence the psychological wellbeing of older adults, promoting better mental health (21, 22). Regarding social factors, community-based home care services can effectively improve the psychological health of older adults (18, 23). The socioeconomic status of older adults has a significant impact on their mental health, and this effect can be partially explained by the mediating role of social participation (24). Additionally, enjoying social pension insurance can alleviate psychological burdens and provide a protective effect on their mental health (25, 26).

In summary, the academic research on factors affecting the psychological health of older adults is extensive, covering various aspects such as individual, family, and societal influences. However, in today's highly developed internet era, the lifestyle of older adults is rapidly changing, and they are increasingly using the internet, becoming part of the digital age. Therefore, exploring the impact of the internet on the psychological health of older adults has become increasingly important, emerging as a new focal point in the study of factors affecting the psychological health of older adults both domestically and internationally. Consequently, this paper will focus on analyzing the relationship between internet use and the psychological health of older adults.

### 2.2 Study on the impact of internet use on the mental health of older adults

Currently, research on the impact of internet use on the mental health of older adults, both domestically and internationally, is divided into two distinctly different perspectives, leading to varying research conclusions: One perspective posits that internet use can significantly enhance the mental health of older adults and has a protective effect on their psychological wellbeing. After receiving internet training, older adults experience a significant improvement in their mental health levels (27). Compared to older adults who do not use the internet, those who do have a lower incidence of depression (5, 28). Moreover, the higher the frequency of internet use, the greater the proficiency, and the more diverse the functions used, the healthier the mental state of the older adults (29). Internet use can help older adults gain a greater sense of security, alleviate feelings of helplessness, generate positive emotions, enhance community belonging, and improve their quality of life (1, 30). Additionally, older adults can acquire more health knowledge and information through the internet (31), which plays a role in their self-management of distress, thereby enhancing their mental health levels (32). Furthermore, from the perspective of specific internet functionalities, some scholars have found that informal online leisure activities (such as leisure and entertainment) help improve the mental health of older adults (2), whereas formal online productive activities (such as learning and work) do not have a significant positive impact on their mental health (33).

Another perspective suggests that internet use has adverse effects on the mental health of older adults. Older adults who use the internet excessively tend to experience greater feelings of loneliness (34, 35). The more they rely on the internet to obtain information, the poorer their mental health tends to be (2). As a “relatively vulnerable group” in the realm of internet use, older adults may be negatively affected emotionally by the mixed quality of information and the proliferation of fraudulent advertisements online, which can harm their mental health (36, 37). Additionally, among older internet users, there are many “silver-haired screen addicts”, and this internet addiction or “digital obsession” can also harm their mental health to some extent (38, 39).

So, what impact does internet use ultimately have on the mental health of older adults? Activity theory and disengagement theory may offer different explanations for this. Activity theory posits that staying active is an important component of later life. The higher the activity level of older adults, the stronger their social participation and adaptability, leading to increased life satisfaction and positive effects on physical and mental health. The internet, as an important platform for activities, can help older adults reintegrate into society, expand their social circles, and enhance social connections, thereby improving mental health (40). In contrast, disengagement theory suggests that as people age, their various abilities gradually decline, making it difficult for them to continue fulfilling their original social roles, and thus they should gradually withdraw from society. However, the advent of the internet has enabled many older adults to remain socially active through online platforms and even take on new social roles. While this continued social participation may delay the disengagement process, it could also increase the psychological and physiological burdens on older adults, thereby negatively impacting their mental health (41). This further highlights the complex interplay between technology use and the wellbeing of older adults.

### 2.3 The mediating role of social networks

In the process of seeking answers to related questions, scholars have increasingly focused on the mechanisms by which the Internet affects the mental health of older adults. Both domestic and international scholars have primarily explored the mediating role of social networks (social capital) between the Internet and the mental health of older adults. Furthermore, some scholars have categorized the two opposing effects of the Internet on the mental health of older adults through social networks into the “network enhancement benefit theory” and the “presence substitution benefit theory” (42).

The “network enhancement benefit theory” posits that the use of the Internet can help older adults expand their existing social networks, establish more social interactions and connections, and acquire new human capital, cultural capital, and social trust, which in turn positively impacts their mental health. Specifically, the use of the Internet by older adults can enhance their interactions with their children and broaden their family and friend networks, thereby reducing their sense of loneliness (35, 43) and promoting their mental health. Other scholars have pointed out that Internet use plays a positive role in increasing the social capital of older adults, enabling them to expand or maintain social connections, enhance social bonds, and increase access to information, thereby reducing their sense of loneliness (44). Additionally, older adults can overcome the barriers of time and distance through the Internet, which helps reduce the cost of social interactions, increase the frequency of social interactions, alleviate loneliness, positively affect their emotions, and maintain a better psychological state (16, 34).

In contrast, the “presence substitution effect theory” suggests that the use of the Internet, especially excessive use by older adults, can encroach upon time for other activities, leading to obstacles in offline participation (39). This can, to varying degrees, reduce the older adults' social participation, interpersonal interactions, and sense of community belonging, thereby exerting a negative impact on them (9, 38). Additionally, excessive Internet use by older adults may decrease their likelihood of using traditional information media, encroach upon their sleep time, and increase the likelihood of holding virtual financial products, thereby introducing uncertain risk factors that could harm their mental health (45, 46).

### 2.4 Research questions

Existing research on the relationship between internet use and the mental health of older adults has identified several key areas that warrant further investigation. First, while many studies have examined the general impact of internet use, the effects of internet use frequency and specific purposes remain underexplored. Second, the mediating role of social networks—particularly the distinct contributions of family and friend networks—in this relationship requires deeper examination. Third, the differential impacts of various internet purposes (e.g., information acquisition, social interaction, entertainment) on mental health have not been fully elucidated.

This study aims to address these areas through the following research questions, using two-wave panel data from the 2018 and 2020 China Longitudinal Aging Social Survey (CLASS):

Direct and Mediated Effects of Internet Use: Does internet use directly impact the mental health of older adults? Do family and friend networks mediate the relationship between internet use and mental health?

Frequency of Internet Use: Does increased frequency of internet use positively affect the mental health of older adults? Do family and friend networks mediate the relationship between internet use frequency and mental health?

Differential Impacts of Internet Purposes: Do different purposes of internet use (e.g., information acquisition, social interaction, entertainment) have distinct impacts on the mental health of older adults Do family and friend networks mediate the relationship between specific internet purposes and mental health?

By examining these questions, this study seeks to provide a comprehensive understanding of how variations in internet use behaviors influence the mental health of older adults through social networks.

## 3 Methods

### 3.1 Data source

The China Longitudinal Aging Social Survey (CLASS) is a national, longitudinal survey implemented by Renmin University of China. It employs a stratified multi-stage probability sampling method to conduct surveys across 28 provinces in China. The CLASS project relies on the China Social Survey Network (CSSN) for field data collection. Participating units form multiple survey teams to conduct face-to-face interviews and read questionnaires to respondents. The survey has been completed in four waves: 2014, 2016, 2018, and 2020, with effective sample sizes of 11,511, 11,471, 11,470, and 11,398 individuals aged 60 and above, respectively. These samples adequately represent the older adult population in China. Given that CLASS only inquired in detail about internet usage (including whether they use the internet, frequency of use, and purposes of use) in the 2018 and 2020 surveys, this study selects data from these 2 years to form a two-time-point longitudinal survey dataset, resulting in an original tracking sample of 9,162 individuals. The inclusion and exclusion criteria of this study were: (1) deleting the missing values of basic demographic and economic characteristics (age, sex, education level, marital status, ADL, pension, income etc.); (2) delete samples with missing data due to reasons such as death or loss of follow-up during the follow-up investigation. Retain respondents who have completed the assessment of depressive symptoms in the initial interview survey and at least one follow-up survey.

### 3.2 Variable measurement

#### 3.2.1 Dependent variable

Mental health was the dependent variable for this paper. There has been extensive discussion in academia regarding the measurement of mental health in older adults, with depression levels considered the primary indicator for assessing the mental health status of older adults. Based on this, the present study continues to use the depression score of older adults as a proxy indicator of their mental health, measured using the 9-item Center for Epidemiologic Studies Depression Scale (CES-D) revised by Silverstein et al. (47). In the CLASS questionnaire, the item “Next, we would like to know about your mood in the past week?” consists of nine questions, each with three response options: 0 (“Never”), 1 (“Sometimes”), and 2 (“Frequently”). For three specific questions—“Did you feel very good in the past week?”, “Did you feel that your days went well in the past week?”, and “Did you find life full of enjoyment (interesting things) in the past week?”—reverse scoring is applied. The scores for all nine questions are summed to generate the final depression score for older adults, with a range from 0 to 18. The score is then standardized, with higher scores indicating worse mental health among older adults. Additionally, in this study, the Cronbach's alpha coefficient for the depression scale across the full sample entering the regression analysis was 0.7239, while for the subsample of internet users, the Cronbach's alpha coefficient was 0.6912. These values indicate relatively acceptable internal consistency (reliability) of the scale.

#### 3.2.2 Independent variable

Internet use was the independent variable for this paper, including three indicators: “whether to use the internet,” “internet usage frequency,” and “internet usage purpose.” The indicators “whether to use the internet” and “internet usage frequency” are generated from the CLASS questionnaire item “Do you use the internet?” Respondents who answer “never use the internet” are assigned a value of 0; those who answer “a few times a year,” “at least once a month,” “at least once a week,” and “every day” are assigned a value of 1, generating the “whether to use the internet” variable. Additionally, based on the frequency of responses to this question, the “internet usage frequency” variable is generated, which includes three levels: “low frequency,” “medium frequency,” and “high frequency.” The “internet usage purpose” indicator is primarily derived from the CLASS questionnaire item “What do you generally do when you use the internet?” The 11 options under this item are consolidated into three functional categories: interpersonal communication (voice/video chat, text chat), leisure and entertainment (listening to music/radio/watching videos, playing games), and information acquisition (shopping, reading news, browsing various articles/information other than news, transportation, health management, investment and financial management, learning and training). If any function within the interpersonal communication category is used, it is assigned a value of 1; if none are used, it is assigned a value of 0, thus generating the “interpersonal communication” variable. Similarly, if any function within the entertainment and leisure category is used, it is assigned a value of 1; if none are used, it is assigned a value of 0, generating the “leisure and entertainment” variable. If any function within the information acquisition category is used, it is assigned a value of 1; if none are used, it is assigned a value of 0, generating the “information acquisition” variable.

#### 3.2.3 Mediator variable

Social network, which includes two indicators: family network and friends network. The Lubben Social Network Scale is used to measure the social networks of older adults, encompassing both family and friends networks. The CLASS data uses the social network scale (LSNS-6) developed by Lubben et al. to measure the social network status of older adults. The scale includes the following questions: “How many family members/relatives can you meet or contact at least once a month?” “How many family members/relatives can you trust to confide in your personal matters?” “When you are in need, how many family members/relatives can provide you with assistance?” “How many friends can you meet or contact at least once a month?” “How many friends can you trust to confide in your personal matters?” “When you are in need, how many friends can provide you with assistance?” The response options are “None,” “1,” “2,” “3–4,” “5–8,” and “9 or more,” which are assigned values of 0, 1, 2, 3, 4, and 5, respectively. The total score from all the questions is used as an indicator of the social network status of older adults, with higher scores indicating greater social support. Among these, the family network score is calculated by summing the scores of the first three questions, while the friend network score is calculated by summing the scores of the last three questions. The range of values for both is 0–15 points. Additionally, in this study, the Cronbach's alpha coefficient for the family network was 0.7981, and that for the friend network was 0.8293. Both coefficients indicate high internal consistency, suggesting that the measurements of these two network dimensions are reliable.

#### 3.2.4 Control variables

Following the existing literature, this paper selects the following control variables: gender, age, urban-rural residency, education level, marital status, political affiliation, personal income, self-rated health, pension status, presence of chronic diseases, financial support from children, and basic activities of daily living (ADL).

The specific descriptions of variables and details of descriptive statistics are presented in Table 1.

**Table 1:** Descriptive statistics of variables.

**Variables**	**Year 2018 (mean)**			**Year 2020 (mean)**			**Variable description**
	**Overall (*N* = 9,162)**	**Online (*N* = 1,800)**	**Non-online (*N* = 7,362)**	**Overall (*N* = 9,162)**	**Online (*N* = 2,250)**	**Non-online (*N* = 6,912)**	
**Dependent variable**							
Depression	8.231	7.625	8.383	6.864	5.748	7.235	CES-D scale depression index
**Independent variables**							
Internet usage (proportion)	—	19.65	80.35	—	24.56	75.44	Yes = 1, No = 0
Internet usage frequency	1.368	1.368	—	1.471	1.471	—	Low = 1, Medium = 2, High = 3
Interpersonal communication	0.878	0.878	—	0.920	0.920	—	Yes = 1, No = 0
Leisure and entertainment	0.538	0.538	—	0.583	0.583	—	Yes = 1, No = 0
Information acquisition	0.729	0.729	—	0.762	0.762	—	Yes = 1, No = 0
**Mediator variables**							
Family network	7.360	7.775	7.259	7.500	7.500	7.499	The higher the score, the better.
Friendship network	6.391	7.105	6.216	6.713	7.047	6.604	The higher the score, the better.
**Control variables**							
Gender	0.503	0.523	0.498	0.504	0.524	0.497	Male = 1, Female = 0
Age	75.051	71.384	75.948	75.064	71.447	76.242	Respondent's actual age
Urban	0.566	0.794	0.510	0.537	0.722	0.476	Urban = 1, Rural = 0
Education	1.431	1.914	1.313	1.430	1.797	1.311	Primary or below = 1, Middle school = 2, High school or above = 3
Marry	0.722	0.839	0.693	0.744	0.851	0.709	Yes = 1, No = 0
Political affiliation	0.032	0.056	0.026	0.034	0.060	0.026	Yes = 1, No = 0
Self-rated health	2.287	2.421	2.255	2.289	2.467	2.231	Unhealthy = 1, Average = 2, Healthy = 3
Chronic diseases	0.739	0.761	0.733	0.790	0.791	0.790	Yes = 1, No = 0
ADL	0.522	0.254	0.587	0.749	0.287	0.900	Higher scores indicate worse outcomes
Ln-income	8.186	8.764	8.029	8.874	9.066	8.752	Logarithm of personal income
Pension	0.753	0.848	0.729	0.781	0.837	0.763	Yes = 1, No = 0
Children financial support	0.890	0.907	0.886	0.917	0.930	0.913	Yes = 1, No = 0

The data is sourced from the CLASS panel data for the periods of 2018 and 2020.

### 3.3 Statistical analysis

Due to the inherent limitations of cross-sectional data in most real-world studies, which often result in substantial unobserved individual heterogeneity leading to omitted variable bias, this paper utilizes longitudinal panel data from the CLASS surveys conducted in 2018 and 2020. This approach allows for an examination of the relationships between internet usage, frequency of internet use, and the purposes of internet use among older adults and their mental health. By adopting this methodology, the study effectively mitigates the issues related to omitted variable bias stemming from unobservable factors such as personality and preferences. The longitudinal nature of the data further allows for a better understanding of the dynamic effects over time, controlling for time-invariant individual characteristics through fixed effects. This helps ensure that the observed relationships are not driven by unobserved heterogeneity. The model is specified as follows:


$$\begin{array}{l} Y_{it}=\beta_{0}+\beta_{1}internet_{it}+\beta_{2}X_{it}+u_{i}+\varepsilon_{it} \end{array}$$

Here, *Y*_*it*_represents the depression score of individual *i* at time *t*, with higher scores reflecting poorer mental health. internetit is the primary explanatory variable, encompassing whether older adults use the internet, their frequency of internet use, and the purposes of internet use. *X*_*it*_ includes control variables that influence mental health, such asphysiological, economic, and social factors. *u*_*i*_ represents individual fixed effects, and ε_*it*_denotes the error term.

In selecting the regression model, this paper conducted the following relevant tests: (1) the F-test, which helps determine whether to choose a pooled regression or a fixed effects model; (2) the LM test, which assists in deciding between a pooled regression and a random effects model; and (3) the Hausman test, which aids in choosing between fixed effects and random effects models. The specific results of the *p*-values for the F-test, LM test, and Hausman test are all 0.0000. Consequently, this study ultimately selects the fixed effects model (FE) for regression analysis.

Furthermore, to explore the mechanisms through which internet usage impacts the mental health of older adults, this study includes social networks (family network and friendship network) as mediating variables and employs the Bootstrap method to test the significance of their coefficients. In the academic literature, there are generally two main approaches for testing mediation effects: the first is the causal step regression method proposed by Baron and Kenny (48), known as the three-step method; the second is the coefficient product method, which includes the Sobel test and the Bootstrap method introduced by Preacher and Hayes (49). However, the Sobel test is generally not utilized by researchers as the primary method for testing mediation effects; rather, it is used as a verification or supplementary explanation for the stepwise regression method. The validity of the Sobel test is predicated on the assumption that the product of coefficients follows a normal distribution, which is often not the case in research, resulting in significant drawbacks. The Bootstrap test also verifies mediation effects by directly examining the product of coefficients; however, it does not assume that the product follows a normal distribution, making its results more reliable compared to the previous two methods. Therefore, in this study, the Bootstrap method is employed to test the mediating role of social networks (family and friendship networks) in the relationship between internet usage and mental health. Specifically, 5,000 resamples are used to construct the sampling distribution of the mediation effect, and the 95% confidence intervals are computed. If the confidence interval does not include zero, the mediation effect is considered statistically significant. All data analyses in this paper were conducted using Stata 17.

## 4 Results

### 4.1 Descriptive statistics

From Table 1, it can be observed that, both in 2018 and 2020, the older adult population using the internet had the lowest average depression scores compared to the overall older adult population and the non-internet users. This indicates that, based solely on the descriptive statistical analysis, the older adult population using the internet exhibited lower levels of depression and better mental health. Additionally, from a temporal perspective, both the overall sample and older adults, whether internet users or non-internet users, showed significantly better mental health in 2020 than in 2018. For instance, compared to older adult internet users in 2018, the average depression score for older adult internet users in 2020 decreased by 1.877, reflecting a noticeable improvement in their mental health.

From the data in Table 1, it is evident that although the proportion of older adults who do not use the internet remains significantly higher than that of those who do, the internet usage among older adults increased significantly between 2018 and 2020. Specifically, the proportion of internet users among older adults rose from 19.65% in 2018 to 24.56% in 2020. Moreover, both the frequency of internet use and the diversity of online activities among older adults showed an upward trend. This shift reflects how older adults are gradually adapting to technological developments, increasingly choosing to use the internet. Additionally, in terms of sample distribution, the average age of the older adults surveyed is around 75 years old, but the average age of internet users is ~71, which is 4 years younger than the overall sample, indicating that younger older adults are more likely to use the internet. The majority of older adults have an elementary-level education, reflecting a generally lower level of educational attainment. Furthermore, over 70% of internet users among older adults reside in urban areas, and most older adults receive pension benefits and financial support from their children. In terms of social network support, older adults generally have broader family network support, but the use of the internet is more effective in expanding their friendship networks.

### 4.2 Selection: fixed effects analysis of the impact of internet usage on the mental health of older adults

#### 4.2.1 The impact of internet usage on the mental health of older adults

This section first analyzes the relationship between internet usage among older adults and their mental health. A stepwise regression approach is adopted, incorporating control variables, the primary independent variable (internet usage), and mediating variables sequentially, with the results presented in Table 2. Model 1a serves as the baseline regression model, illustrating the relationship between control variables and the mental health of older adults. The regression results indicate that, the results indicate that male older adults, those living in urban areas, married individuals, those with better self-rated health, and those receiving pensions have lower levels of depression and better mental health compared to other types of older adults.

**Table 2:** Regression results of internet usage and mental health of older adults.

**Variables**	**Model 1a**	**Model 1b**	**Model 1c**
	**Depression**	**Depression**	**Depression**
Male	−1.483^***^	−1.574^***^	−1.099^***^
Age	−0.053	−0.053	−0.020
Urban	−0.414^***^	−0.433^***^	−0.662^***^
Education			
Junior high school	0.977^***^	0.968^***^	0.611
High school and above	−0.368	−0.575	−0.534
Married	−0.343^**^	−0.342^**^	0.097
Communist party member	−0.034	−0.037	0.078
Self-rated health			
Fair	−0.195^*^	−0.150	−0.219^**^
Good	−0.391^***^	−0.322^***^	−0.340^***^
Chronic illness	−0.141^*^	−0.117	0.061
ADL	0.011	0.005	0.041^**^
Income	−0.051	−0.057^*^	−0.064^**^
Pension	−0.447^***^	−0.433^**^	0.037
Economic support from children	−0.141	−0.103	0.055
Internet usage		−0.440^***^	−0.258^***^
Year 2020			−0.532^***^
Internet usage in 2020			−0.146^**^
Sample	9,739	9,739	9,739
*R* ^2^	0.032	0.051	0.260
Adjusted *R*^2^	0.030	0.049	0.259

Data sourced from the same Table 1; ^*^p < 0.05, ^**^ *p* < 0.01, ^***^ *p* < 0.001.

Model 1b incorporates internet usage and shows that internet use is associated with a 0.44 unit reduction in depression scores, suggesting its positive impact on mental health. Additionally, after introducing internet usage, individual income levels become significant, highlighting the role of economic factors in mental wellbeing.

Building on Model 1b, Model 1c adds an interaction term for “internet usage and year” to explore how internet usage affects the mental health of older adults across different years. The results show that, compared to 2018, the mental health of older adults improved in 2020, with average depression scores decreasing by 0.532 units. Regardless of the year, internet usage significantly enhances mental health. Further analysis reveals that the positive effect of internet usage on mental health in 2020 is more pronounced than in 2018, suggesting that the impact of internet usage on mental health is not static but has become more significant over time, influenced by changes in social conditions and technology.

#### 4.2.2 The impact of internet usage frequency on the mental health of older adults

To further explore the impact of internet usage behavior characteristics on the mental health of older adults, this section discusses the relationship between internet usage frequency and the mental health of older adults, with regression results presented in Table 3. Model 2a serves as the baseline regression, which includes only control variables. Model 2b builds upon the baseline regression by incorporating the core independent variable of internet usage frequency. Model 2c further adds the interaction term of internet usage and frequency to Model 2b.

**Table 3:** Regression results of internet usage frequency and mental health of older adults.

**Variables**	**Model 2a**	**Model 2b**	**Model 2c**
	**Depression**	**Depression**	**Depression**
Internet usage frequency			
Medium frequency		−0.286	−0.213
High frequency		−0.454^***^	−0.258^***^
Year 2020			−0.531^***^
Medium frequency#2020			−0.147
High frequency #2020			−0.150^***^
Control variables	Control	Control	Control
Sample size	9,739	9,739	9,739
*R* ^2^	0.032	0.051	0.260
Adjusted *R*^2^	0.030	0.050	0.258

Data sourced from the same Table 1; ^*^p < 0.05, ^**^ *p* < 0.01, ^***^ *p* < 0.001.

From Model 2b, we observe that the regression coefficient for high-frequency internet usage is −0.454, which is statistically significant at the 0.001 level, indicating that high-frequency internet usage significantly reduces depression levels among older adults. Specifically, with each unit increase in high-frequency internet usage, the average depression level of older adults decreases by 0.454 units. This finding underscores the crucial role of high-frequency internet usage in enhancing the mental health of older adults.

Similar to Model 1c, Model 2c incorporates the interaction term of “internet usage frequency and year” based on Model 2b. The regression results indicate that, compared to 2018, the depression scores of older adults in 2020 decreased by an average of 0.531 units. Furthermore, regardless of the year, high-frequency internet usage among older adults significantly improves their mental health status. Further analysis reveals that the positive impact of high-frequency internet usage on the mental health of older adults in 2020 is more pronounced than that in 2018.

#### 4.2.3 The impact of internet usage purpose on the mental health of older adults

This study further examines the influence of specific internet usage purposes—interpersonal communication, leisure and entertainment, and information acquisition—on the depression levels of older adults. The regression results are presented in Models 3a, 3b, and 3c in Table 4.

**Table 4:** Regression results of internet usage purposes and mental health of older adults.

**Variables**	**Model 3a**	**Model 3b**	**Model 3c**
	**Depression**	**Depression**	**Depression**
Interpersonal communication	−1.218^***^		
Information retrieval		−0.358^**^	
Leisure and entertainment			−0.375^***^
Control variables	Control	Control	Control
Sample	2,654	2,654	2,654
*R* ^2^	0.077	0.082	0.092
Adjusted *R*^2^	0.072	0.077	0.087

Data sourced from the same Table 1; ^*^p < 0.05, ^**^ *p* < 0.01, ^***^ *p* < 0.001.

The results demonstrate that each of these internet usage purposes has a significant positive impact on the mental health of older adults, with distinct effects. Model 3a shows that the regression coefficient for interpersonal communication is −1.218 (*p* < 0.001), indicating that engaging in online interpersonal communication significantly reduces depression levels among older adults. This highlights its critical role in alleviating loneliness and enhancing psychological wellbeing. Model 3b reveals a regression coefficient of −0.358 (*p* < 0.01) for information acquisition, suggesting that accessing information online helps older adults maintain cognitive engagement and reduce depressive symptoms. Model 3c finds that leisure and entertainment activities, with a regression coefficient of −0.375 (*p* < 0.001), provide older adults with enjoyable experiences that effectively mitigate depressive emotions. These findings underscore the multidimensional positive effects of internet use in improving the mental health of older adults.

### 4.3 Selection: heterogeneity analysis of the impact of internet use on the mental health of older adults

Internet use has a significant impact on the mental health of older adults; however, its effects may vary across different groups of older adults. Therefore, this section will focus on analyzing the differential impacts of whether older adults use the internet, the frequency of internet use, and the purposes of internet use among various gender and urban-rural groups. This will reveal the unique patterns of internet use on mental health across different demographics. The regression results are presented in Tables 5, 6.

**Table 5:** Heterogeneity of the impact of internet use and frequency on the mental health of older adults by gender and urban-rural status.

**Variables**	**Male**	**Female**	**Urban**	**Rural**
	**Depression**	**Depression**	**Depression**	**Depression**
Internet use	−0.454^***^	−0.450^***^	−0.420^***^	−0.587
	(0.095)	(0.093)	(0.069)	(0.325)
Internet use frequency				
Moderate frequency	−0.400	−0.201	−0.171	−0.663^**^
	(0.288)	(0.372)	(0.262)	(0.251)
High frequency	−0.465^***^	−0.463^***^	−0.429^***^	−0.612
	(0.098)	(0.096)	(0.071)	(0.390)
Control variables	Control	Control	Control	Control
Sample	4,929	4,810	6,598	3,141

Data sourced from the same Table 1; ^*^p < 0.05, ^**^ *p* < 0.01, ^***^ *p* < 0.001; Standard errors are in parentheses.

**Table 6:** Heterogeneity of the impact of internet usage purposes on the mental health of older adults by gender and urban-rural status.

**Variables**	**Male**	**Female**	**Urban**	**Rural**
	**Depression**	**Depression**	**Depression**	**Depression**
Interpersonal communication	0.153	−1.579^***^	−1.501^***^	8.703^***^
	(0.527)	(0.297)	(0.355)	(0.000)
Information acquisition	−0.020	−0.689^***^	−0.398^**^	8.703^***^
	(0.174)	(0.175)	(0.127)	(0.000)
Leisure and entertainment	−0.289^*^	−0.468^***^	−0.419^***^	1.873^***^
	(0.139)	(0.140)	(0.101)	(0.000)
Control variables	Control	Control	Control	Control
Sample	1,367	1,287	2,301	353

Data sourced from the same Table 1; ^*^p < 0.05, ^**^ *p* < 0.01, ^***^ *p* < 0.001; Standard errors are in parentheses.

Table 5 reports the differences in the effects of internet use and internet usage frequency on the mental health of older adults across different genders and urban-rural contexts. The regression results indicate significant heterogeneity in the impact of internet use on the mental health of older adults based on gender and urban-rural distinctions. First, from a gender perspective, internet use has a significant positive effect on the mental health of both male and female older adults. Specifically, the regression coefficient for the relationship between internet use and depression levels among male older adults is −0.454 (*p* < 0.001), while for female older adults, it is −0.450 (*p* < 0.001). This suggests that internet usage significantly reduces depression levels in older adults, with little difference between males and females. However, when considering internet usage frequency, the results show that high-frequency internet use has a significant negative impact on the depression levels of both male and female older adults (males: −0.465, *p* < 0.001; females: −0.463, *p* < 0.001), while the impact of moderate-frequency internet use on depression levels is statistically insignificant. This indicates that frequent internet use may be an effective means of alleviating depressive symptoms among older adults.

Second, from an urban-rural perspective, internet use has a significant negative impact on the depression levels of urban older adults (−0.420, *p* < 0.001), while its effect on rural older adults is statistically insignificant (−0.587, *p* > 0.05). This discrepancy may be related to differences in internet penetration rates and usage habits between urban and rural areas. Further analysis of the impact of internet usage frequency on the mental health of older adults in urban and rural settings reveals that high-frequency internet use has a significant negative effect on the depression levels of urban older adults (−0.429, *p* < 0.001), while its impact on rural older adults is statistically insignificant (−0.612, *p* > 0.05). However, moderate-frequency internet use has a significant negative impact on the depression levels of rural older adults (−0.663, *p* < 0.01), while its effect on urban older adults is statistically insignificant. This indicates that rural older adults may benefit more from moderate internet activities, whereas urban older adults require more frequent internet usage to significantly improve their mental health.

Furthermore, Table 6 reports the differences in the impact of various internet usage purposes on the mental health of older adults across different genders and urban-rural settings. The findings indicate that the effects of internet usage purposes on the depression levels of older adults exhibit significant heterogeneity based on gender and geographic location. First, from a gender perspective, there are notable differences in how different internet usage purposes affect the depression levels of male and female older adults. For male older adults, the impact of internet usage for interpersonal communication on their depression levels is statistically insignificant (0.153, *p* > 0.05), whereas the purposes of information acquisition and recreational activities have a negative effect on their depression levels, with the latter showing a significant impact (−0.289, *p* < 0.05). This suggests that male older adults can significantly reduce their depression levels by utilizing the internet for information acquisition and engaging in recreational activities. In contrast, female older adults exhibit a significant negative impact from all three internet usage purposes—interpersonal communication, information acquisition, and recreational activities—especially in the areas of interpersonal communication (−1.579, *p* < 0.001) and recreational activities (−0.468, *p* < 0.001). This indicates that female older adults experience a more pronounced positive effect on their mental health through online interpersonal communication and recreational activities.

Secondly, from an urban-rural perspective, there are also significant differences in how various internet usage purposes affect the depression levels of older adults in urban and rural settings. For urban older adults, the purposes of interpersonal communication (−1.501, *p* < 0.001), information acquisition (−0.398, *p* < 0.01), and recreational activities (−0.419, *p* < 0.001) all have a significant negative impact on their depression levels. This suggests that urban older adults can significantly improve their mental health by engaging in these online activities. In contrast, rural older adults exhibit a significant positive impact from all three internet usage purposes (interpersonal communication: 8.703, *p* < 0.001; information acquisition: 8.703, *p* < 0.001; recreational activities: 1.873, *p* < 0.001), indicating that these activities may actually increase their depression levels. This result may reflect rural older adults' maladjustment to internet usage or the influence of other socioeconomic factors.

### 4.4 Link: analysis of the mediating effects of family networks and friend networks

This section aims to use the Bootstrap method to verify the mediating effects of family networks and friend networks in the relationship between internet usage and the mental health of older adults. Through path analysis and mediation effect testing, we can gain a deeper understanding of how internet usage influences the mental health status of older adults through social networks. Table 7 shows that internet usage has a significant indirect effect on the mental health of older adults through family networks and friend networks. Specifically, the mediating effect through family networks is −0.016, while the mediating effect through friend networks is −0.011, both of which pass the significance test. This indicates that internet usage can improve the mental health of older adults by enhancing their social support networks. Notably, the mediating effect of friend networks (4.46%) is slightly higher than that of family networks (2.25%), which may reflect the importance of friend networks in the mental health of older adults. In the analysis of internet usage frequency, the results show that both medium and high frequency of internet usage can also have a significant positive impact on mental health through family networks and friend networks. The mediating effect of family networks is −0.003, while that of friend networks is −0.006, with their respective contributions to mediation effects being 2.23 and 4.38%, both showing significant significance. This further supports the positive impact of internet usage frequency on the mental health of older adults, particularly through the enhancement of social networks.

**Table 7:** Mediation effect test of family network and friend network (bootstrap).

**Variables**	**Mechanism**	**Mediating effect**	**Proportion (%)**	**95% CI**	**Significance**
Internet usage	Family Network	−0.016	2.25	(−0.026, −0.008)	Yes
	Friend Network	−0.011	4.46	(−0.016, −0.007)	Yes
Internet usage frequency	Family network	−0.003	2.23	(−0.005, −0.001)	Yes
	Friend network	−0.006	4.38	(−0.008, −0.003)	Yes
Interpersonal communication	Family network	−0.023	6.28	(−0.039, −0.008)	Yes
	Friend network	−0.014	3.94	(−0.032, 0.001)	No
Leisure and entertainment	Family network	0.017	7.18	(0.008, 0.027)	Yes
	Friend network	0.006	2.66	(0.001, 0.013)	Yes
Information acquisition	Family network	0.012	10.01	(0.003, 0.022)	Yes
	Friend network	0.008	6.89	(0.001, 0.017)	Yes

In the analysis of internet usage purposes, the mediating effect of interpersonal communication through family networks on depression levels is −0.023, which is significant within the 95% confidence interval (−0.039, −0.008). However, the mediating effect through friend networks is −0.014, which does not reach significance. This indicates that older adults can use the internet for interpersonal communication to expand their family networks, thereby promoting improvements in their mental health status.

Further observation of the mediating effects of family networks and friend networks in the context of leisure and entertainment, as well as information retrieval purposes, reveals that the indirect effect coefficients for social networks in relation to leisure and information retrieval purposes and the mental health of older adults do not include zero in their confidence intervals. However, the direction of the indirect effect coefficients is contrary to that of the direct effect coefficients. This suggests that family networks and friend networks exhibit a significant masking effect between leisure and information retrieval purposes of the internet and the depression scores of older adults—while leisure and information retrieval purposes can effectively reduce the depression levels of older adults, they may also compress the family and friend networks of older adults, thereby negatively impacting their mental health.

## 5 Discussion

This study explores the complex mechanisms through which internet usage influences the mental health of older adults, emphasizing the multidimensional disparities shaped by sociocultural contexts, economic backgrounds, and the dynamic interactions between older adults and digital technologies. While internet use generally contributes to lower depression levels among older adults (β = −0.44, *p* < 0.001), this finding is also consistent with conclusions drawn from previous studies (29, 40, 50), the moderating effects of urban-rural differences, gender disparities, and socioeconomic status show that the mental health benefits of technology are not universally experienced, but are deeply embedded in structural inequalities.

First, the limited or even negative mental health benefits observed in rural older adults suggest that the issue extends beyond the traditional “digital divide” centered around internet access. Our results reveal that older adults internet usage frequency correlates positively with depression levels (e.g., leisure activities: β = 1.873, *p* < 0.001). This paradox can be analyzed through several dimensions: (1) Cultural Conflicts in Technological Practices. Rural societies rely heavily on physical co-presence as a social bond. Online interactions, in contrast, fail to provide the emotional support associated with physical proximity (51). Data show that rural seniors primarily engage in passive consumption of digital content (e.g., livestream viewing, 72%), while urban peers actively engage in building new social networks via algorithm-driven interest groups (e.g., short-video platforms) (52). This disparity aligns with Appadurai's “technoscape” theory, where rural populations, acting as “spectators” of globalized digital content, may feel existentially isolated due to their inability to participate meaningfully in digital spaces (53). (2) Infrastructure-Digital Literacy Interplay. Despite the increase in internet penetration in rural areas (a 3.3% increase from 2018 to 2020), 32% of rural respondents reported unstable connectivity, compared to 8% in urban areas (54). These fragmented technological experiences may lead to frustrations, reducing the perceived efficacy of technology (55). Moreover, lower educational attainment among rural seniors limits their capacity to use complex digital functions (e.g., online health consultations), relegating technology to a source of entertainment rather than substantive social support. (3) Intergenerational Relationships as Mediators. Although rural older women may alleviate loneliness through video calls with children, this practice may reinforce a form of “digital filial piety,” where physical visits are reduced in favor of virtual interactions, indirectly exacerbating emotional deprivation. This transformation in intergenerational relationships highlights the fragility of rural family structures and the paradoxical role of technology in fostering empowerment while deepening social disconnection.

Second, central to understanding these dynamics is the role of social networks as mediators in the relationship between internet usage and mental health. We draw upon social capital theory to elucidate how the internet affects mental wellbeing through two opposing paradigms: the “network enhancement benefit theory” and the “presence substitution benefit theory” (56, 57). The “network enhancement benefit theory” suggests that internet usage can strengthen social connections and increase social support, thereby improving mental wellbeing. Our findings support this perspective, as we observed that internet usage, particularly high-frequency internet activities focused on interpersonal communication, significantly improved the mental health of older adults. This effect was mediated through both family and friend networks, underscoring the role of social connections in enhancing the benefits of internet usage. The mediating effects through these networks were found to be significant, with family networks contributing 2.23% and friend networks contributing 4.38%. This suggests that the internet, when used for social purposes, can strengthen social ties and alleviate depressive symptoms among older adults.

However, we also observed a counterbalancing “masking effect” related to the use of the internet for leisure and information retrieval purposes. According to the “presence substitution benefit theory,” this effect occurs when online interactions, although beneficial in terms of reducing depressive symptoms, may lead to a reduction in face-to-face interactions, potentially causing social isolation (9, 58). In our study, the indirect effects of family and friend networks for leisure and information-seeking purposes were significant, but they appeared to reduce the size of social networks. This supports the notion that the internet's role in mental health is complex, as certain uses of the internet can diminish offline interactions, potentially negating the positive effects of social support. This masking effect, observed primarily in the context of leisure and information retrieval purposes, warrants further investigation to better understand how the internet's multifaceted roles influence the mental wellbeing of older adults.

Third, the moderating effect of income on the mental health benefits derived from internet use (a 0.64% unit improvement per 10% increase in income) highlights the role of technology in reinforcing social hierarchies. Wealthier older adults have access to premium services (e.g., paid online courses, telemedicine) and can optimize their content consumption through personalized recommendations (e.g., tailored health information), creating a “virtuous technological spiral” (59). Additionally, limited education and economic resources confine low-income seniors to basic internet functions, often resulting in repetitive entertainment consumption (e.g., short videos), which displaces time without building social capital (60). This pattern aligns with Bourdieu's theory of capital conversion, where economic capital is converted into cultural capital through digital engagement, further exacerbating health inequities (61).

We found that older adults with higher annual incomes tend to enjoy better mental health status, suggesting that economic factors play a crucial role in enhancing the benefits of internet usage. This outcome reaffirms previous research indicating the intersection of socioeconomic status and mental health (62–64). As such, any efforts aimed at promoting digital engagement among older adults should also consider the socioeconomic context, ensuring that disadvantaged groups are provided with the necessary resources and support to benefit fully from digital technologies.

## 6 Limitations

Although this paper systematically analyzes the significant impact of Internet use on older adults' mental health and the mediating role of social networks, it has certain limitations. First, the study mainly relies on panel data from 2018 to 2020, which covers a relatively short time span and thus cannot fully capture the long-term effects of Internet use on older adults' mental health. Second, while the fixed-effects model controls for endogeneity issues caused by time-invariant factors, it cannot eliminate endogeneity issues arising from time-varying factors. Finally, the study uses the CES-D (Center for Epidemiologic Studies Depression Scale) as a measure of mental health, which mainly focuses on depressive symptoms. While CES-D is a widely used instrument for assessing depressive symptoms, it may not fully capture the broader spectrum of mental health, such as anxiety, wellbeing, or overall psychological resilience. This limitation restricts the study's ability to assess the full impact of Internet use on older adults' mental health. Future research should further differentiate the specific content and modes of Internet use to gain a more comprehensive understanding of its effects on the mental health of older adults, and incorporate other mental health measures, such as the GHQ−12 or WHO−5, for a more comprehensive assessment.

## 7 Conclusion

This study explores the impact of internet use on depression among older adults, highlighting variations based on gender, urban-rural residency, and the purposes of internet engagement. The findings indicate that internet activities centered on social interaction contribute to improved mental health by strengthening social ties, whereas internet use primarily for information-seeking or leisure may limit the expansion of social networks.

These patterns can be better understood through the “network enhancement benefit theory” and the “presence substitution benefit theory”. The social functions of the internet help older adults maintain and expand their social networks, increase social capital, and reduce loneliness, thereby fostering mental wellbeing. However, when internet use is predominantly focused on information consumption or leisure activities, particularly in an immersive manner, it may reduce the frequency of interactions with family and friends, weakening the supportive role of real-world social networks. Furthermore, according to the “displacement hypothesis”, online activities may encroach upon time spent on offline social interactions, diminishing opportunities for meaningful in-person engagement. Additionally, “cognitive load theory” suggests that excessive exposure to overwhelming or distressing information may impose a psychological burden, exacerbating stress and negatively affecting emotional wellbeing. As such, the mental health impact of internet use is not uniformly positive but rather contingent upon usage patterns, content quality, and the balance between digital and real-world social interactions.

These findings underscore the necessity of targeted digital inclusion initiatives, particularly for socially isolated individuals and those in rural areas, to maximize the mental health benefits of internet use. Policymakers and social service organizations should focus on fostering inclusive digital environments that facilitate older adults' integration into the online sphere while encouraging internet use in ways that enhance social interaction and support systems.

Moreover, this study holds important practical and clinical implications. Digital literacy training programs emphasizing social engagement tools can play a critical role in alleviating loneliness among older adults. It is also crucial to promote a balanced approach to online and offline interactions to prevent social isolation resulting from excessive digital dependence. Ensuring equitable access to digital resources for economically disadvantaged and rural populations remains essential. Furthermore, integrating digital skills training into mental health interventions, particularly for those at high risk of depression and loneliness, can enhance overall wellbeing and facilitate a smoother transition into the digital era.

## Data Availability

The raw data supporting the conclusions of this article will be made available by the authors, without undue reservation. For those interested in accessing the raw CLASS data, requests should be directed to the official website: http://class.ruc.edu.cn/.
